# Practical Challenges of Switching and Cross-Titration Between Incretin-Based Therapies in Type 2 Diabetes and Obesity: A Narrative Review

**DOI:** 10.3390/jcm15218295

**Published:** 2026-10-24

**Authors:** Jana Makuc, Miodrag Janić

**Affiliations:** 1 Topolšica Hospital, Topolšica 64, 3326 Topolšica, Slovenia; 2 Community Health Centre Ravne na Koroškem, Ob Suhi 11, 2390 Ravne na Koroškem, Slovenia; 3 Faculty of Medicine, University of Ljubljana, Vrazov trg 2, 1000 Ljubljana, Slovenia; miodrag.janic@kclj.si; 4 Department of Endocrinology, Diabetes, and Metabolic Diseases, Division of Internal Medicine, University Medical Centre Ljubljana, Zaloška 7, 1000 Ljubljana, Slovenia

**Keywords:** GLP-1 RA, dual GIP/GLP-1 receptor agonist, tirzepatide, switching, cross-titration, type 2 diabetes mellitus, obesity, treatment response heterogeneity

## Abstract

Glucagon-like peptide-1 (GLP-1) receptor agonists and the dual glucose-dependent insulinotropic polypeptide (GIP)/GLP-1 receptor agonist tirzepatide have transformed the treatment of type 2 diabetes mellitus (T2DM) and obesity. Although these agents share an incretin-based mode of action, they differ in structure, receptor pharmacology, pharmacokinetics, formulation, and titration schedule, and the individual treatment response is highly heterogeneous. Switching between agents occurs in roughly one in four to one in three patients in real-world practice, yet no standardized protocol exists: recommendations rest largely on expert consensus, and no official dose-equivalence chart has been issued. This narrative review critically evaluates evidence for switching and cross-titration between incretin-based therapies; summarizes, insofar as they inform switching decisions, the pharmacological diversity and response heterogeneity that motivate switching; and translates the evaluated evidence into a stepwise practical switching algorithm, developed by the authors as an evidence-informed expert synthesis that has not been formally validated. Special populations, persistence, discontinuation, weight regain, and the emerging pipeline of multi-agonists and oral small molecules are addressed where they modify switching decisions. Until the evidence matures, individualized reason-specific transitions that preserve the continuity of incretin-based therapy offer the most defensible framework for practice.

## 1. Introduction

Incretin-based therapies, glucagon-like peptide-1 (GLP-1) receptor agonists (GLP-1 RAs) and the dual glucose-dependent insulinotropic polypeptide (GIP)/GLP-1 receptor agonist tirzepatide, have transformed the pharmacological management of type 2 diabetes mellitus (T2DM) and obesity. Beyond glycemic control, members of this class reduce major adverse cardiovascular events (MACE) in patients with T2DM [1,2,3,4,5] and in individuals with overweight or obesity and established cardiovascular disease but without diabetes [6]. They achieve weight reductions that were previously attainable only with metabolic surgery: approximately 15% of baseline body weight (BW) with semaglutide 2.4 mg [7] and approximately 21% with tirzepatide 15 mg [8], with superiority of tirzepatide 15 mg over semaglutide 2.4 mg demonstrated head-to-head in obesity [9] and in T2DM [10]. The therapeutic landscape continues to expand, with additional molecules, indications and formulations entering routine practice. Furthermore, recent phase 3b trials have expanded this therapeutic ceiling, demonstrating that a higher maintenance dose of once-weekly semaglutide 7.2 mg can achieve mean body weight reductions of 18.7% in adults with obesity and 13.2% in those with concurrent T2DM [11].

Despite a shared incretin-based mode of action, these agents are not pharmacologically interchangeable. They differ in molecular structure, receptor engagement, pharmacokinetics (PK), and pharmacodynamics (PD), formulation and titration schedule. Their individual treatment response is heterogeneous, spanning glycemic and weight outcomes and the doses required to achieve them. Up to approximately 30% of patients fail to reach clinically significant weight loss with a given agent [12], and response to one molecule does not reliably predict response to another [13]. Against this background, switching between agents has become common in real-world practice: a retrospective real-world analysis reported switching between GLP-1 RAs in 25.5% of patients with T2DM [14]; population-level data document switching frequencies approaching 30%, 14.3% of patients switching two or more times, and a rising trend over recent years [15]; and switching occurs in approximately 20–29% of patients treated for obesity without diabetes [16,17] and switching, discontinuation and reinitiation frequently alternate within the same treatment episode [18].

However, no standardized, evidence-based protocol governs these transitions. Existing recommendations are derived from expert consensus rather than dedicated randomized switching trials [19,20,21]. Manufacturers or regulators have not issued an official dose-equivalence chart, and pharmacometric analyses have formally demonstrated that equal milligram doses are not clinically equivalent across molecules [22]. Only recently has direct evidence begun to emerge. Randomized SURPASS-SWITCH trial showed that switching from dulaglutide to tirzepatide outperforms dose escalation of the incumbent agent [23]. Prospective switch data support a direct switch of patients non-naïve in GLP-1 RA to 5 mg of tirzepatide without re-titration [24] and a randomized pilot study demonstrated that flexible and slowed titration markedly reduces withdrawal from gastrointestinal treatment [25]. Also, model-based simulations have characterized PK overlap and cross-titration trajectories during transitions [26,27].

This narrative review focuses on the critical assessment of switching and cross-titration between incretin-based therapies. Its primary objective is to evaluate the available switching evidence, from randomized trials, prospective and real-world switch studies, and pharmacometric models, and to determine how much it supports specific transition strategies in clinical practice. Secondary objectives are (i) to summarize, insofar as they directly inform switching decisions, the pharmacological differences between agents and the heterogeneity of treatment response that constitute the rationale for switching; (ii) to translate the appraised evidence and published consensus into a practical stepwise switching algorithm; and (iii) to identify evidence gaps and priorities for future research. Pharmacological diversity, responder phenotypes, and emerging therapies are therefore discussed selectively and only where they directly affect switching decisions.

## 2. Literature Search and Evidence Selection

This narrative review is based on targeted searches of PubMed/MEDLINE and Google Scholar, complemented by screening of ClinicalTrials.gov and manual review of the reference lists of included publications. Regulatory information was retrieved directly from the European Medicines Agency (EMA), the US Food and Drug Administration (FDA) and the UK Medicines and Healthcare products Regulatory Agency (MHRA) websites and from the current Summaries of Product Characteristics and prescribing information for each agent [28,29,30,31]. Searches covered the period from database inception to 1 September 2026 and combined the following principal terms and their variants: “GLP-1 receptor agonist”, “glucagon-like peptide-1”, “tirzepatide”, “GIP/GLP-1”, “switch”, “switching”, “transition”, “cross-titration”, “dose equivalence”, “titration”, “treatment response”, “non-response”, “persistence”, “discontinuation” and “weight regain”. Only publications in English were considered. Eligible sources comprised randomized controlled trials and their post hoc and pooled analyzes, observational and real-world studies, pharmacometric and model-based simulation studies, systematic reviews and meta-analyzes, case reports and case series, expert consensus statements and practical guidance documents, and regulatory documents. Preprints were eligible but are explicitly identified as such in the text and are interpreted with caution. Titles and abstracts were screened by both authors, full texts of potentially relevant publications were assessed, and disagreements were resolved by consensus. In total, 119 publications were included in this review. In prioritizing evidence for the practice-oriented recommendations, randomized data were favored over observational data, observational data over pharmacometric modeling, and modeling over expert consensus. Regulatory documents and product labeling were treated as the definitive source for dosing, titration and indication statements.

The populations covered are adults with T2DM and adults with overweight or obesity treated with incretin-based therapies. Pediatric populations, type 1 diabetes, and indications other than glycemic control or weight management are outside the scope of this review. The treatment transitions covered are (i) switches between GLP-1 RAs, (ii) switches from a GLP-1 RA to the dual GIP/GLP-1 receptor agonist tirzepatide or vice versa, (iii) transitions between injectable and oral incretin formulations, and (iv) the cross-titration and dose-conversion strategies used during such transitions. Initiation of incretin therapy in treatment-naïve patients and switches between incretin-based and non-incretin drug classes are not evaluated. The outcomes evaluated are glycemic control (HbA1c), body weight, treatment persistence, gastrointestinal and other adverse events, and weight regain after discontinuation, which is considered the counterfactual to switching.

## 3. Pharmacological Diversity of Incretin-Based Therapies

Incretin-based therapies now comprise several GLP-1 RAs and a dual GIP/GLP-1 receptor agonist, tirzepatide, most of which are approved for the treatment of T2DM and, for selected agents, obesity. Although these molecules share an incretin-based mode of action, they differ substantially in molecular structure, receptor pharmacology, PK/PD, formulation, and dose regimen (Table 1), and these differences plausibly contribute to highly individualized treatment response [32]. Understanding this diversity is a prerequisite for rational switching, because a change of molecule alters receptor engagement, exposure profile, and administration conditions simultaneously.

### 3.1. Molecular Structure, Receptor Pharmacology and Pharmacokinetics

Native GLP-1 is inactivated within minutes by dipeptidyl peptidase-4 (DPP-4), so all approved agents incorporate stabilizing modifications [33]. Exenatide and lixisenatide are exendin-4-based analogs with short half-lives (approximately 2 to 4 h) requiring injection once or twice-daily, while liraglutide and semaglutide are lipidated human GLP-1 analogs whose fatty-acid side chains mediate albumin binding, extending half-lives to approximately 13 h and one week, respectively [32,33,34]. Tirzepatide, a 39-amino-acid peptide derived largely from the GIP sequence and carrying a C20 fatty acid moiety, has a half-life of approximately five days [37]. These differences are directly relevant to the switch in three ways. First, half-life determines the pharmacological carry-over during a transition: when moving between long-acting agents, the departing molecule remains pharmacologically active for one to two weeks, creating an overlap window. Second, PK differences translate into PD differences, short-acting agents durably delay gastric emptying, whereas long-acting agents induce partial tachyphylaxis of this effect so the tolerability profile can change across a switch [32,38,39]. Third, tirzepatide is a biased GLP-1 receptor agonist with an imbalanced potency profile across its two receptors, and its GIP component contributes materially to its metabolic efficacy [35,40]. Together with reported associations between common variation in *GLP1R* and differential response [41], this provides a mechanistic basis for why a patient may respond to one molecule but not to another.

### 3.2. Formulations, Dosing Regimens and Administration Conditions

Daily injectable options (exenatide twice daily, lixisenatide, and liraglutide) coexist with weekly injectables (exenatide extended-release, dulaglutide, semaglutide, and tirzepatide) and with the first orally administered peptide of the class. Oral semaglutide is co-formulated with the absorption enhancer sodium N-(8-[2-hydroxybenzoyl]amino) caprylate (SNAC), which transiently elevates local gastric pH, protects peptide from proteolytic degradation, and promotes transcellular absorption through the gastric epithelium [42]. This mechanism imposes stringent administration conditions, intake in the fasting state with no more than approximately 120 mL of water and a delay of at least 30 min before food, beverages or other oral medication, and even under these conditions absolute bioavailability remains low (approximately 1%) with substantial within- and between-subject variability [43,44]. These constraints are directly relevant when converting patients between oral and injectable incretin therapies, because exposure achieved with a nominal milligram dose differs by more than an order of magnitude between routes. A second oral agent, the non-peptide small-molecule GLP-1 RA orforglipron, was approved by the US FDA in April 2026 for chronic weight management and authorized by the UK MHRA in August 2026 for both weight management and T2DM, following the phase 3 ATTAIN and ACHIEVE programmes [30,31,45,46]. A marketing authorization application covering obesity and T2DM remains under EMA evaluation at the time of writing. Unlike oral semaglutide it requires no absorption enhancer and no fasting or water restrictions, properties that make it an attractive oral landing option in switching strategies.

All product labels prescribe gradual dose increase, typically in four-week steps, to mitigate gastrointestinal adverse events (AEs) (Table 1). Real-world titration, however, frequently diverges from the label. In a nationwide Danish cohort that initiated semaglutide for weight management, most patients did not increase according to the recommended schedule and only a minority reached the maintenance dose of 2.4 mg [47]; in a population-based study in the UK in T2DM, persistence was suboptimal and dose escalation was frequently delayed [48]. This divergence complicates the interpretation of “non-response” in practice, because a proportion of apparently inadequate responses may reflect under-dosing rather than true pharmacological resistance.

### 3.3. Efficacy and Cardiovascular Outcomes

Efficacy within the class forms a gradient, and this gradient is the main driver of efficacy-motivated switches. Escalating once-weekly semaglutide from 1.0 mg to 2.0 mg provided additional HbA1c and body-weight reductions in SUSTAIN FORTE [49]; tirzepatide outperformed semaglutide 1.0 mg at both endpoints in SURPASS-2 [10] and semaglutide 2.4 mg in the head-to-head SURMOUNT-5 trial in obesity [9]; and semaglutide 7.2 mg extended the dose–response further in STEP UP and STEP UP T2D [11,46]. Weight loss is consistently attenuated in patients with T2DM (approximately 10% with semaglutide 2.4 mg in STEP 2 and approximately 15% with tirzepatide in SURMOUNT-2) [50,51], which must be considered when judging response before and after a switch. Cardiovascular outcome data further differentiate the molecules and can motivate or constrain agent choice: liraglutide [1], subcutaneous semaglutide [3], dulaglutide [2], and oral semaglutide [4] each reduced MACE; semaglutide 1.0 mg also reduced major kidney events in FLOW [52]; and semaglutide 2.4 mg reduced cardiovascular events in obesity without diabetes in SELECT [6]. Most relevant to switching, tirzepatide was non-inferior to dulaglutide 1.5 mg for MACE in SURPASS-CVOT while producing substantially greater reductions in body weight and HbA1c [5], which is directly reassuring for transitions from dulaglutide to tirzepatide in patients with T2DM and established cardiovascular disease. The same molecule may therefore be dosed, titrated, and judged successful by different criteria in T2DM (HbA1c attainment, cardiorenal protection) than in obesity (magnitude and durability of weight loss).

Three features of Table 1 are directly relevant to switching. First, molecules differ in the number of titration steps and in the proportion of patients who will ever need the top maintenance dose, so a “failed” low dose of one agent does not predefine failure of another. Second, the approved indication matters practically: liraglutide and semaglutide carry separate T2DM and obesity labels with different maximum doses, whereas tirzepatide uses an identical titration ladder for both, so the target maintenance dose must be chosen against the indication being treated rather than the molecule alone. Third, the class has expanded beyond peptides: orforglipron, a small non-peptide molecule approved in the United States and the United Kingdom in 2026 (with an EU application pending) whose half-life supports once-daily oral dosing without food or water restrictions, is expected to simplify injectable-to-oral transitions in practice [31,45,46].

## 4. Heterogeneity of Treatment Response

Even under the controlled conditions of pivotal trials, and more so in routine care, the response to GLP-1 RAs and tirzepatide is markedly variable between individuals [53]. This heterogeneity that spans BW and glycemic outcomes, the doses required to achieve them, and the speed with which the trajectories separate provides the clinical rationale for switching strategies.

### 4.1. Responder Phenotypes in Obesity and T2DM

**Figure 1:**
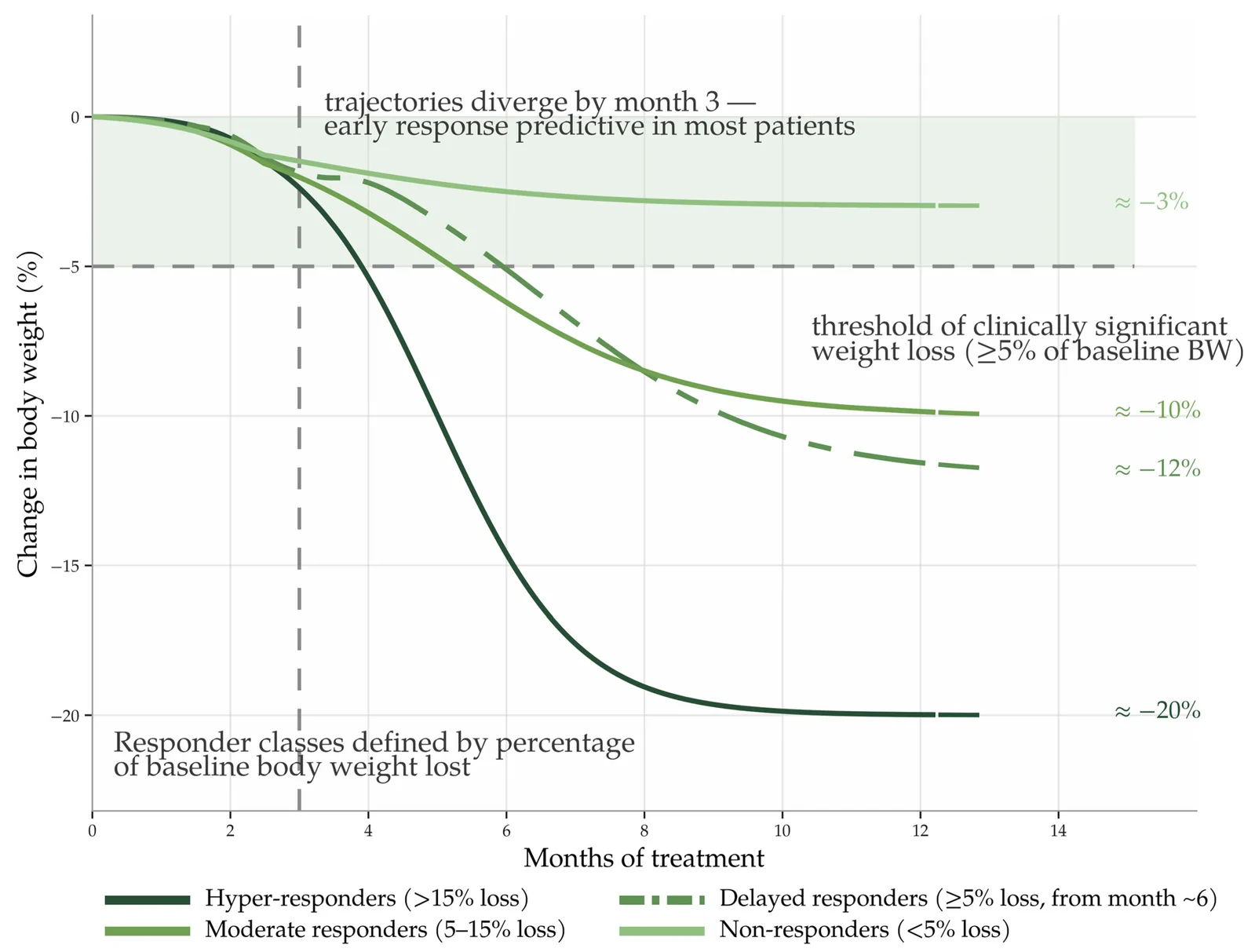
Schematic of body weight (BW)-loss trajectories by responder phenotype during incretin-based therapy. Curves represent hyper-responders (>15% loss), moderate responders (5–15%), delayed responders (clinically meaningful loss emerging ~month 6), and non-responders (<5%). A vertical marker at month 3 denotes the typical divergence point for early response prediction [53,56]. The delayed-responder trajectory highlights why this month-3 assessment must incorporate parallel metabolic improvements (e.g., glycemic control) to avoid premature switching. The shaded band indicates the ≥5% threshold for clinically significant weight loss, which ~30% of patients fail to reach [12], underscoring the need for early reassessment.

For obesity pharmacotherapy, patients can be stratified by the percentage of baseline BW lost: non-responders (<5%), moderate responders (5–15%) and hyper-responders (>15%) [53,54] (Figure 1). Response definitions have recently been formalized in a dedicated synthesis [55]: non-response is pragmatically defined as less than 5% total body-weight loss after 16–24 weeks on the maximum tolerated dose, with modest (5–10%), good (10–20%), and super-response (≥20%) strata, and glycemic response defined as an HbA1c reduction of at least 0.5–1.0 percentage points. Distinct “glycemic super-responder” and “weight super-responder” phenotypes may coexist in T2DM, consistent with the multidimensional response patterns documented in the Diabetes-Patienten-Verlaufsdokumentation (DPV) register [56]. The most granular characterization of hyper-responders currently derives from a large real-world analysis available so far only as a preprint, which should therefore be interpreted with appropriate caution [54]. Complementary registry data from T2DM show that response is not a single dimension: in the prospective DPV register, only 14% of patients achieved marked reductions in both glycated hemoglobin (HbA1c) and BW, while 35.7% responded predominantly in HbA1c and 7.4% predominantly in weight, the remainder qualifying as non-responders on either axis [56]. This multidimensionality of response gives rise to striking discordant phenotypes in clinical practice. For example, some patients exhibit profound weight loss while concurrently experiencing a paradoxical worsening of HbA1c. Mechanistically, such discordant responses highlight the dual pathways of incretin action: the central anorexigenic effect remains intact, driving substantial weight loss, whereas the glycemic benefit is abolished if endogenous beta-cell reserve is severely depleted (such as in unrecognized latent autoimmune diabetes or advanced beta-cell exhaustion in T2DM). In such cases, the failure to improve HbA1c should not be misinterpreted as a global failure of the molecule, but rather as an indication of exhausted beta-cell function requiring concomitant insulin therapy [57].

Overall, up to approximately 30% of patients fail to achieve clinically significant (≥5%) weight loss with incretin-based pharmacotherapy [12]. Counterbalancing this, dose requirements are also heterogeneous: real-world titration studies indicate that only approximately one quarter of patients ever reach the maximum tested doses of semaglutide or tirzepatide [16,47], implying that “adequate trial of therapy” is itself an individualized concept.

Beyond the standard categories of non-responders and hyper-responders, emerging clinical evidence identifies a distinct subgroup of “delayed responders.” These individuals exhibit minimal weight loss during the first 3 to 4 months of treatment but eventually achieve clinically meaningful weight reduction by month 6 and beyond. This delayed trajectory is most frequently observed in patients with pronounced baseline insulin resistance or long-standing T2DM, where incretin therapies initially work to reverse metabolic dysfunction, such as reducing fasting insulin levels and normalizing glycemia, before substantial adipose tissue reduction occurs. Recognizing this phenotype is crucial to prevent the premature discontinuation of a highly effective therapy [53,58].

### 4.2. Early Trajectories Predict Late Outcomes

Responder phenotypes are not only definable retrospectively; they emerge early. In real-world obesity cohorts, BW-loss trajectories for non-responders, moderate responders, and hyper-responders diverge within approximately the first three months of treatment, allowing the expected response class to be inferred at that point [54]. A post hoc analysis of SURMOUNT-1 similarly showed that the early BW trajectory under tirzepatide predicts the magnitude of subsequent weight reduction, supporting the early response as a pragmatic decision node [59]. Consistent with this framework, a structured monitoring schedule with early assessments at weeks 4 and 8 and a formal response evaluation by week 16 on the maximum tolerated dose has been proposed, with patients who fail to achieve at least 5% body-weight loss or a meaningful HbA1c reduction flagged for reassessment, including dose escalation, behavioral intensification, or a switch of agent [55]. Building on this proposal and on the early-trajectory data cited above, the present review applies a single staged follow-up pathway consistently throughout: weeks 4 and 8 serve as early safety, gastrointestinal-tolerability, and glucose checks, including adjustment of concomitant medication; week 12 (approximately month 3) constitutes the initial efficacy assessment and the primary decision node, based on HbA1c, body-weight trajectory, adherence, dose exposure, and tolerability; week 16 is a conditional rather than routine assessment, performed when dose titration is still ongoing at month 3 or when the month-3 response is equivocal; and month 6 is reserved for the reassessment of patients provisionally classified as delayed responders. These observations carry an important implication for switching: waiting for a formal plateau at the maximum dose before reconsidering therapy may prolong exposure to an ineffective agent, while structured assessment around month 3 could identify candidates for dose escalation, adherence support, or a change of molecule, provided that label-recommended titration has genuinely been completed, which real-world data suggest is not always the case [47,48]. However, the month-3 decision node must be applied with caution to accommodate delayed responders. While a lack of weight reduction by month 3 generally predicts poor long-term weight outcomes in obesity, clinicians must evaluate parallel metabolic markers. Patients with limited early weight loss (less than 5% of baseline body weight) at month 3 who nevertheless show meaningful concurrent metabolic improvement, defined as an HbA1c reduction of at least 0.5–1.0 percentage points, a substantial reduction in insulin requirements, or both, may be provisionally classified as delayed responders [53,55,58]. In these patients, extending structured observation up to month 6 before initiating a switch is clinically justified, as the weight response may simply be delayed by ongoing metabolic repair. The outcomes to be evaluated at month 6 are the emergence of clinically meaningful weight loss, the durability of the glycemic improvement, and continued tolerability and adherence [53,58].

### 4.3. Predictors of Response: Sex, Genetics, Microbiota and Diabetes Status

Despite intense interest, few reproducible predictors of response have emerged. Female sex is the only robust clinical predictor of a greater weight response [16,53,60]. Common genetic variation in peptidyl-glycine alpha-amidating monooxygenase (PAM) and *GLP1R* has been associated with differential GLP-1 RA response [41,61,62,63]. Gut microbiota composition correlates with responsiveness, although evidence remains associative [64] and T2DM itself attenuates weight reduction [65], consistent with the attenuated weight loss observed in STEP 2 and SURMOUNT-2 relative to their non-diabetic counterparts. Post hoc analyzes of SURPASS-4 indicate that the baseline determinants of glycemic and weight responses to tirzepatide overlap only partially, reinforcing the multidimensional nature of response [66].

Taken together, individual response cannot yet be predicted with clinically useful precision [12,55]. For the purposes of switching, the practical conclusions are threefold: an inadequate response should prompt verification that label-recommended titration was genuinely completed before the molecule is judged ineffective; response is molecule-specific rather than class-specific, so failure of one agent neither predicts nor precludes response to another [13]; and structured trial-and-error, including switching between molecules with different receptor pharmacology, remains a rational, evidence-compatible strategy for the minority of patients whose initial response is inadequate [55].

## 5. Why and When to Switch

The decision to switch between GLP-1 RAs and GIP/GLP-1 receptor agonist tirzepatide is driven by four broad, frequently overlapping categories of triggers: inadequate therapeutic response or plateau, AEs, constraints of access and cost, and less commonly patient preference regarding formulation or dosing frequency [19,20,67]. Understanding which trigger applies is clinically consequential, because it determines the choice of successor agent, the starting dose, and the speed of titration.

### 5.1. Inadequate Response and Plateau

Given the marked heterogeneity of the response, the failure to achieve glycemic or weight-related goals with one agent does not preclude a meaningful response to another. A recent case report documented a patient with class 2 obesity who was a clear non-responder to tirzepatide but subsequently lost weight on semaglutide, illustrating that the response is molecule-specific rather than class-specific [13]. Real-world studies in T2DM consistently show that patients switching between once-a-week GLP-1 RAs achieve further reductions in HbA1c and BW, although smaller than those seen in treatment-naïve individuals [68,69]. As noted from the DPV register data, many apparent “non-responders” actually respond in only one domain [56], so a switch may rationally target the unmet domain rather than global inefficacy.

A pharmacological plateau, typically emerging after 12–18 months of treatment as compensatory mechanisms counterbalance weight loss, should be distinguished from a true non-response, because escalating or switching at the plateau has a different expected yield than switching an early non-responder [13,53]. Randomized evidence that switching can outperform intensification of the current agent comes from the SURPASS-SWITCH trial, in which adults with T2DM inadequately controlled on dulaglutide achieved significantly greater HbA1c and BW reductions when switching to tirzepatide than when increased to the highest dose of dulaglutide [23]; subgroup analyzes confirmed the consistency of this benefit across clinically relevant strata [70].

Alternatively, an inadequate response or a plateau on standard maintenance doses may be addressed by in-class dose escalation before considering a switch of the molecule. The STEP UP and STEP UP T2D trials demonstrated that tripling the semaglutide dose from 2.4 mg to 7.2 mg at week 20 without additional intermediate titration steps yields further clinical benefits. This escalation resulted in an additional estimated treatment difference of −3.1% in body weight reduction versus the 2.4 mg dose in patients without diabetes, suggesting that a higher dose can push beyond an early efficacy ceiling [46].

### 5.2. Adverse Events

Gastrointestinal (GI) AEs, nausea, vomiting, diarrhea, and constipation, are the most frequent treatment-limiting effects of incretin-based therapies and a leading trigger of switching or discontinuation in both trial and real-world settings [67,71,72]. Because these effects are dose-dependent and are partially attenuated over time through tachyphylaxis, switching to an alternative molecule, a slower titration schedule, or a different formulation may restore tolerability without abandoning the class [38,71]. For tirzepatide specifically, transition to a selective GLP-1 RA has been suggested as a tolerability strategy when gastrointestinal toxicity is dose-limiting, reflecting the contribution of the GIP component to its adverse-effect profile [55]. With the introduction of higher-dose regimens, new adverse event profiles are also emerging that could influence switching decisions. Specifically, dysaesthesia (altered skin sensation, such as hyperaesthesia, burning sensation, or sensitive skin) was reported significantly more frequently with semaglutide 7.2 mg compared to the 2.4 mg dose or placebo. These events occurred in 18.9% of patients with T2DM and 22.9% of patients without diabetes. Although these events were predominantly mild to moderate, generally resolved without dose reduction, and rarely led to permanent discontinuation, their rapid onset following dose escalation represents a novel tolerability consideration that may prompt dose reduction or a switch to an alternative agent in clinical practice [11,46].

Rare but serious AEs require a different logic: acute pancreatitis temporally associated with tirzepatide has been reported during or shortly after the transition from another GLP-1 RA [73], and meta-analytic data confirm small but real increases in pancreatitis and the risk of gallbladder disease [74,75]. In such scenarios, immediate discontinuation is mandatory. If incretin-induced acute pancreatitis is confirmed, restarting the same agent or switching to any alternative incretin-based therapy is strictly advised against by clinical guidelines due to the presumed class-wide nature of this risk, necessitating a definitive shift to non-incretin treatment modalities [76].

### 5.3. Access, Cost, Shortages and Compounded Products

A substantial proportion of real-world switches are non-voluntary. Global shortages of semaglutide and tirzepatide between 2022 and 2025 forced abrupt transitions, dose interruptions, and therapeutic substitutions, for which practical strategies, but no trial evidence have been published [77,78]. Population-level data from Poland document how switching patterns between 2018 and 2025 tracked market availability and reimbursement policy rather than clinical indications [15]. Cost and insurance coverage are equally potent drivers. In a large survey of adults who discontinued GLP-1 RA therapy, cost and loss of coverage were among the most frequently cited reasons [79]. Shortages also fueled the use of compounded and counterfeit products; the American Diabetes Association (ADA) has advised against the use of compounded GLP-1 RAs due to dosing errors and uncertain content [80], and commentaries have documented the global scale and regulatory gaps of this market [81,82]. When patients arrive on such products, clinicians should manage the transition to an approved agent as a formal de novo switch with re-titration.

## 6. Switching and Cross-Titration Strategies

The principal studies informing switching and cross-titration are summarized at the study level in Table 2, which specifies the population, prior treatment and dose, transition regimen, comparator where applicable, follow-up, principal outcomes, and key limitations of each source.

### 6.1. Consensus Principles and Their Evidence Base

Practical recommendations for switching between GLP-1 RAs and tirzepatide have been formulated primarily for the T2DM [19,21], with a subsequently published practical guide extending them to dual agonists [20]. It must be explicitly stated that these recommendations originate from expert consensus rather than from dedicated randomized switch trials, although they are indirectly informed by switch studies and clinical-trial titration experience [83,84,85]. Their core principles, adaptable to obesity management, are as follows:

- Prior to switching, contraindications and special warnings must be reviewed;
- The patient’s history of AEs, particularly GI ones, must be assessed;
- Titration should be individualized;
- When selecting the initial dose and the titration speed, the duration of previous GLP-1 RA treatment and the dose of the prior medication should be considered;
- In principle, the new medication should be initiated at its lowest dose to assess tolerability, although titration may be accelerated in selected cases;
- If the switch is prompted by AEs, titration should proceed more slowly;
- The new medication should be administered on the day the next dose of the original medication would normally be due [19,20,21].

These principles provide a safety-oriented default; where they should be deliberately modified, in particular, regarding the starting dose, is informed by the studies reviewed below.

### 6.2. Timing the Switch and Overlapping Half-Lives

Because the agents involved have long half-lives, approximately five days for tirzepatide and about one week for semaglutide, both active compounds are simultaneously present in the circulation during the initial transition period, even when the new agent is administered when the next dose of the previous one is due [32,37,97]. A model-based simulation of the transition from semaglutide or dulaglutide to tirzepatide confirmed the transient PK overlap and predicted that the glycemic and weight effects of tirzepatide would still follow the expected trajectory of the new agent, supporting a direct, no-washout transition [27]. However, additive PD effects during overlap can transiently increase the likelihood of GI AEs, and vigilance is warranted for rare, serious events; a case of acute pancreatitis in temporal association with a transition to tirzepatide [73,74].

### 6.3. Re-Titration Versus Direct Switch at Higher Doses

The conservative default, restarting at the lowest dose of the new agent, is challenged by accumulating evidence in patients with GLP-1 RA experience. In a prospective study of participants with T2DM on stable GLP-1 RA treatment who switched directly to 5 mg of tirzepatide without re-titration, glycemic control improved further (mean HbA1c change −0.43%) with additional BW reduction (−2.15 kg) over the first 12 weeks, GI AEs were mostly mild, and only about 2% discontinued because of AEs [24]. Real-world data from Japan corroborate the feasibility of direct switches to tirzepatide at non-initiation doses [87], and observational studies of switching between once-weekly GLP-1 RAs report preserved tolerability and continued efficacy in pretreated patients [68,69]. A hospital case series has likewise documented pragmatic dose-conversion approaches with acceptable outcomes [88]. Model-based analyzes provide a pharmacological rationale for these observations: exposure–response simulations indicate that prior GLP-1 RA treatment confers tolerance that permits higher starting doses of the successor agent [26,27], and randomized intra-class switch trials show maintained or improved efficacy after transition [85,86]. Collectively, these data suggest that GLP-1 RA-experienced, tolerability-proven patients can often start the new agent above its nominal initiation dose, whereas AE-driven switchers and patients with treatment gaps should re-titrate from a low dose [20,21].

### 6.4. Dose Equivalence

There is no direct milligram equivalence between GLP-1 RA and tirzepatide, and consequently no official conversion chart has been issued by manufacturers or regulators. PK/PD simulation of the switch from semaglutide to dulaglutide formally demonstrated that equal milligram doses are not clinically equivalent between molecules [22]; conversely, a model-based meta-analysis has derived exposure- and response-based clinical dose equivalences that may approximate comparative potency [98]. Based on available studies, a practical guide has proposed an equivalent-dose table to smooth transitions while preserving glycemic control [20]. In the absence of endorsed guidance, unofficial online conversion charts and dosing “calculators” of unknown provenance proliferate. Their use represents a risk of unregulated-information analogous to that documented for compounded and counterfeit incretin products, unverified dose content reaching patients and clinicians without quality control [80,81,82]. Clinicians should explicitly discourage reliance on such tools and anchor conversions in peer-reviewed data and product labeling.

### 6.5. Flexible and Slowed Titration

When a dose becomes ineffective but further escalation triggers disruptive AEs, flexible titration offers a third path between stagnation and discontinuation. In the open-label randomized pilot study “Gradual Titration of Semaglutide Results in Better Adherence to Treatment and Fewer Adverse Events”, a 16-week flexible titration regimen allowing intermediate dose increments reduced GI-related AEs to 2% compared with 19% under the label-recommended 8-week escalation, with equivalent glycemic and weight outcomes [25]. Pooled analyzes confirm that slower escalation materially reduces nausea incidence [99], and the PIONEER 7 trial demonstrated that flexible, adjustment-guided titration of oral semaglutide is effective and well tolerated [100]. Real-world cohorts show that many patients never reach maximal labeled doses yet achieve clinically meaningful weight loss and remain on therapy, supporting individualized, sometimes deliberately submaximal, dosing [16,47]. This drive for titration granularity has fueled the emerging clinical practice of “microdosing” (or fractional dosing). By exploiting the dose-counter mechanics of multi-dose injection pens (commonly referred to as “click counting”), patients and clinicians intentionally administer sub-therapeutic or highly incremental doses. Recent clinical literature highlights microdosing as a pragmatic strategy to overcome severe gastrointestinal intolerance and retain patients on therapy who would otherwise discontinue due to adverse events [101]. However, this practice is strictly off-label and carries inherent risks. Patient safety organizations caution that manual fractional dosing lacks manufacturer validation for dose accuracy and significantly increases the risk of administration errors [102]. Despite these safety and regulatory concerns, the growing prevalence of microdosing strongly underscores the unmet clinical need for delivery devices that accommodate finer, highly individualized titration options [25,80].

### 6.6. Switching Between Injectables and Oral Formulations

Transitions between injectable and oral incretin therapies involve distinct constraints. Oral semaglutide depends on co-formulation with the absorption enhancer SNAC and requires strict fasting administration with limited water and a post-dose waiting period, conditions that produce high PK variability [42,43,44]. In SWITCH-SEMA 2, switching from DPP-4 inhibitor to oral semaglutide improved glycemic control and weight, supporting oral initiation after non-incretin therapy [89]; extension data from the PIONEER program show that switching from sitagliptin to oral semaglutide yields additional improvements in glycemic control and body weight [90]. Importantly, formal label-based switching guidance has begun to appear. The current EU product information for oral semaglutide includes an explicit conversion table for patients switching from subcutaneous semaglutide (0.5 mg once weekly → 4 or 9 mg; 1 mg once weekly → 9 or 25 mg; 2 mg once weekly → 25 or 50 mg orally once daily), with the oral agent started one week after the last injection [28]; the US prescribing information for semaglutide also specifies direct transitions between 2.4 mg injection and 25 mg oral tablet in both directions [29]. Most recently, the ATTAIN-MAINTAIN trial demonstrated that patients withdrawn from injectable incretin therapy can maintain weight reduction when switched to oral small-molecule GLP-1 RA orforglipron, further supporting the feasibility of higher-than starting dose transitions to oral agents in pretreated patients [91].

### 6.7. Concomitant Medication Adjustments

Switching to a more potent agent alters the risk profile of background therapy. After transition to tirzepatide, insulin requirements drop substantially; real-world data document rapid and marked reductions in insulin dose within weeks of the switch, and failure to proactively de-escalate insulin or sulfonylureas exposes patients to hypoglycemia [92]. Therefore, consensus and practical guidance recommend anticipatory reduction in insulin secretagogues and insulin, guided by baseline HbA1c and glucose monitoring, at the time of switch, with structured follow-up during the first weeks [20,21,71].

Table 3 illustrates an overarching pattern: there is the strongest evidence for efficacy-driven switches from GLP-1 RA to tirzepatide, where randomized data now support switching over dose escalation of the incumbent agent, and for flexible titration, where a randomized pilot study demonstrated large reductions in AE-related withdrawal. For most other scenarios, AE-driven switches, shortage-driven substitutions and injectable-to-oral transitions, recommendations still rest on expert consensus, observational series, and PK modeling rather than dedicated trials. Clinicians should therefore treat the suggested approaches as structured defaults to be individualized: the trigger for switching the prior dose and duration of therapy, the length of any treatment gap, and the concomitant glucose-lowering medication jointly determine the appropriate starting dose and titration speed. Importantly, the absence of an official dose-conversion chart means that every change should be accompanied by explicit counseling, early follow-up, and documentation of tolerability.

## 7. A Practical Switching Algorithm

**Figure 2:**
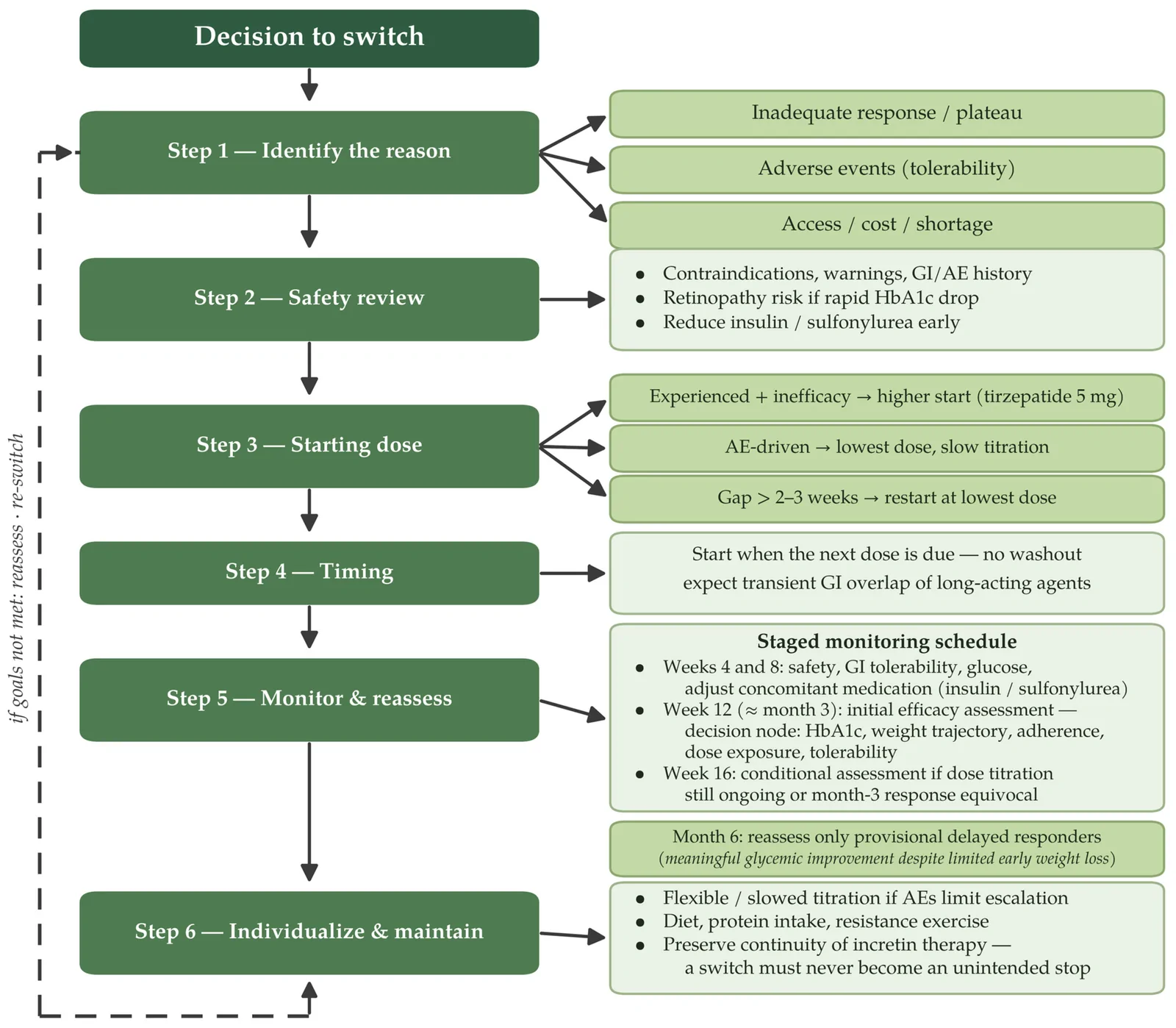
Stepwise clinical algorithm for switching between GLP-1 receptor agonists and the dual GIP/GLP-1 receptor agonist tirzepatide. The transition pathway is determined by the switching trigger (inefficacy, adverse events, or access/shortages) and the baseline clinical risk. Starting-dose selection ranges from direct switches at higher doses for tolerability-proven patients [23,24] to lowest-dose re-titration for those with adverse events or treatment gaps [21,25]. Subsequent steps outline administration timing (no washout), a staged monitoring schedule comprising early safety and tolerability checks at weeks 4 and 8, an initial efficacy assessment and decision node at week 12 (approximately month 3), a conditional week-16 assessment when dose titration is ongoing, and a month-6 reassessment reserved for provisional delayed responders, and flexible titration. This framework synthesizes expert consensus [19,20,21] and recent evidence [23,24,25] to safely ensure the continuity of incretin-based therapy. The algorithm is an expert synthesis developed by the authors and has not been formally validated. AE, adverse event; GI, gastrointestinal; GIP, glucose-dependent insulinotropic polypeptide; GLP-1 RA, glucagon-like peptide-1 receptor agonist; HbA1c, glycated hemoglobin.

The following algorithm (Figure 2) operationalizes the consensus principles of Almandoz et al. [19], Jain et al. [21], and the practical guide of Alqifari et al. [20], and extends them in three respects. First, it incorporates randomized switching evidence that was unavailable when those statements were written, most notably SURPASS-SWITCH, which demonstrated the superiority of switching to tirzepatide over escalation of dulaglutide [23,70], and the randomized flexible-titration pilot study [25]. Second, it modifies the consensus default of a universal lowest-dose restart: for GLP-1 RA-experienced, tolerability-proven patients switching for inadequate efficacy, a direct switch at a higher-than-initial dose is supported by prospective and real-world data [24,87,88], whereas the conservative restart is retained for AE-driven switches and treatment gaps. Third, it adds a structured reassessment schedule that the earlier guidance lacked, derived from early-trajectory analyzes [54,59] and responder-phenotype syntheses [55,58]: early safety and tolerability checks at weeks 4 and 8, an initial efficacy assessment and primary decision node at week 12 (approximately month 3), a conditional assessment at week 16 for patients still undergoing dose titration, and a month-6 reassessment reserved for patients provisionally classified as delayed responders. None of these modifications has been prospectively validated, and the algorithm is presented explicitly as an unvalidated, evidence-informed framework. The special clinical situations described in Section 8 modify the execution of Steps 2–5 (perioperative timing, pregnancy planning, older adults, risk of retinopathy, and altered gastrointestinal anatomy after bariatric surgery) and should be consulted whenever they apply.

The algorithm steps do not rest on a uniform evidence base and, therefore, the provenance of each recommendation is made explicit (Table 4). Direct support from dedicated switching studies exists for four elements: efficacy-driven switching from dulaglutide to tirzepatide, which outperformed escalation of the incumbent agent in a randomized trial [23,70]; direct initiation of tirzepatide 5 mg without re-titration in GLP-1 RA-experienced, tolerability-proven patients, supported by prospective switch data [24] and corroborated by real-world series [87,88]; flexible and slowed titration to reduce gastrointestinal attrition, supported by a randomized pilot study [25] and pooled adverse-event analyzes [99]; and injectable-to-oral maintenance transitions, supported by randomized data for orforglipron [91] and by label-based conversion tables for semaglutide [28,29]. Recommendations resting on indirect or modeling evidence include the no-washout timing rule and the expectation of preserved efficacy across transitions (pharmacometric simulations [26,27]) and the warning against milligram-based dose conversion (model-based dose inequivalence [22]). Recommendations resting primarily on expert consensus and author interpretation include the trigger-based classification of switches (Step 1), re-titration from the lowest dose after treatment gaps (Step 3), anticipatory de-escalation of insulin and sulfonylureas (Step 2, indirectly supported by observational data [92]), and the staged monitoring schedule, comprising the month-3 decision node, the conditional week-16 assessment, and the month-6 reassessment of provisional delayed responders (inferred from early-trajectory studies [54,59] and responder-phenotype syntheses [55,58]). Areas of genuine uncertainty, and therefore priorities for validation, include dose conversion between semaglutide and tirzepatide at higher doses, switching after adverse-event-driven discontinuation of a predecessor agent, and transitions involving the newest agents (orforglipron, retatrutide, CagriSema), for which no dedicated switch data exist. The algorithm should accordingly be regarded as an evidence-informed, expert-synthesized framework that has not been validated, in which each recommendation inherits the strength, or the fragility, of the evidence class from which it derives.

**Step 1—Identify the reason for switching.** Classify the trigger as inadequate response or plateau, AE-driven, or access-/shortage-driven, since the reason determines the successor agent, the starting dose and the titration speed [19,21].

**Step 2—Review safety prerequisites and background therapy.** Verify contraindications and warnings, document the AEs history (particularly GI/pancreatitis), reassess retinopathy risk in patients with long-standing T2DM anticipating rapid glycemic improvement, and review concomitant insulin or sulfonylureas, which should be proactively de-escalated with intensified glucose monitoring [20,92].

**Step 3—Choose the starting dose of the new agent.** In GLP-1 RA-experienced, tolerability-proven patients switching for inefficacy, a direct switch at a higher-than-initial dose is reasonable, for tirzepatide, a 5 mg start is supported by prospective switch data and the SURPASS-SWITCH design [23,24]. For AE-driven switches, restart at the lowest dose and slowly titrate [21,25]. After treatment gaps exceeding approximately 2–3 half-lives of the departing agent (about 2–3 weeks for once-weekly agents), manage the patient effectively as treatment-naïve and restart at the lowest dose [20].

**Step 4—Time the transition.** Administer the new agent when the next dose of the previous agent is due; no washout is required, but transient PK overlap of long-acting agents warrants vigilance for additive gastrointestinal effects [21,27].

**Step 5—Monitor and reassess according to a staged schedule.** After the switch, follow-up proceeds through four defined assessment points. (i) Weeks 4 and 8: assess safety, gastrointestinal tolerability, glucose levels, and the need to adjust concomitant medications, particularly proactive de-escalation of insulin or sulfonylureas. (ii) Week 12 (approximately month 3): perform the initial efficacy assessment based on HbA1c, body-weight trajectory, adherence, dose exposure, and tolerability; this is the primary decision node at which the switch is confirmed, the dose is escalated, or therapy is reconsidered [54,59]. (iii) Week 16: perform an additional assessment only when needed, specifically when dose titration was still ongoing at month 3 or when the month-3 response was equivocal. (iv) Month 6: reassess only patients provisionally classified as delayed responders, defined by limited early weight loss (less than 5% of baseline body weight) despite meaningful concurrent metabolic improvement (HbA1c reduction of at least 0.5–1.0 percentage points, substantially reduced insulin requirements, or both); at month 6, evaluate whether clinically meaningful weight loss has emerged, whether the glycemic improvement is maintained, and whether tolerability and adherence support continuation and, if not, proceed to reconsidering or switching therapy [55,58]. Evaluating metabolic and glycemic markers alongside weight at every node avoids prematurely switching delayed responders whose weight loss may lag behind metabolic repair [58].

**Step 6—Individualize titration and adjunctive measures.** Where efficacy plateaus below target, but AEs limit escalation, apply flexible or slowed titration rather than forced escalation or discontinuation of the label-schedule; reinforce dietary, protein-intake and resistance-exercise measures, and prioritize the continuity of incretin-based therapy to avoid weight regain [25,99]. Furthermore, transitioning to a more potent incretin regimen heightens the risk of drug-related malnutrition secondary to profound appetite suppression. Intensive dietary counseling by a multidisciplinary team is recommended to optimize nutritional intake and prevent deficiencies in essential micronutrients, particularly in women of reproductive potential [104].

## 8. Special Populations and Clinical Situations

The decision to switch between incretin-based therapies rarely occurs in vacuum; comorbidities, life circumstances, and planned interventions frequently shape both the indication for switching and its execution. The following subsections address situations in which switching requires a specific modification of the general principles.

### 8.1. Perioperative Management

GLP-1 RAs delay gastric emptying, and retained gastric contents have been documented despite standard fasting, raising concern about pulmonary aspiration during anesthesia [105]. Multi-society guidance recommends an individualized approach that considers dose, indication, and GI symptoms rather than universal discontinuation [105], and the consensus of the Society for Perioperative Assessment and Quality Improvement (SPAQI) advises that most patients on stable weekly regimens may continue therapy, with risk stratification based on the escalation phase and symptom burden [106]. These recommendations have direct implications for the switch: initiating a switch or increasing after one in the weeks preceding elective surgery may place the patient in the highest-risk window, because GI AEs and gastric-emptying effects are most pronounced early after initiating or changing doses [105,106]. Therefore, a planned procedure may be an opportune reason to delay a non-urgent change, and any recent change should be reported to the anesthesia team [106].

### 8.2. Pregnancy Planning and Contraception

Incretin-based therapies are contraindicated in pregnancy, and preconception counseling is essential for women with potential for childbirth [107]. For semaglutide, a washout is recommended approximately two months before planned conception, reflecting its prolonged half-life; shorter washouts apply to agents that act with shorter action [107]. Because substantial weight loss can restore ovulatory function in obesity-related anovulation, unplanned pregnancies are a realistic concern, and effective contraception should be discussed proactively [107]. For oral agents, delayed gastric emptying can theoretically reduce oral contraceptive absorption, and tirzepatide labeling advises a non-oral or additional barrier method for four weeks after starting and each dose increase [107]. When switching is contemplated in a woman of reproductive age, the choice of the successor agent dictates specific contraceptive counseling. Current 2026 consensus guidelines highlight that tirzepatide reduces the bioavailability of oral hormonal contraceptives more significantly than other GLP-1 RAs due to its rapid dose escalation and greater delay in gastric emptying. Consequently, while additional barrier contraception is not required for patients taking semaglutide, dulaglutide, or liraglutide, patients switching from these agents to tirzepatide must be explicitly advised to use a non-oral or barrier method for four weeks after initiation and for four weeks after each dose increase. Furthermore, if a switch is delayed in favor of structured discontinuation prior to conception, the medication should be stopped 4 to 8 weeks before planned conception based on half-lives, or immediately if pregnancy is confirmed [104].

### 8.3. Older Adults and Sarcopenia Risk

Weight loss with incretin-based therapies comprises both fat and lean mass, and older adults, particularly those with frailty or baseline sarcopenia, may be disproportionately vulnerable to clinically significant muscle loss during rapid weight reduction [108]. Evidence specific to switching in this population is lacking; extrapolation supports slower dose escalation, longer intervals between titration steps, resistance exercise, adequate protein intake, and periodic functional assessment [108]. Where a switch is motivated by tolerability in an older patient, restarting at the lowest dose of the new agent with cautious up-titration is prudent, as age-related changes in gastric motility, polypharmacy, and reduced physiological reserve may amplify GI AEs and the risk of dehydration [108].

### 8.4. Pancreatitis, Gallbladder Disease and Retinopathy

Meta-analyzes of randomized trials indicate that the use of GLP-1 RAs is associated with an increased risk of gallbladder and biliary disease, likely related to rapid weight loss and altered gallbladder motility, with an increased risk at higher doses and longer durations [75]. GI AEs, including a small absolute increase in pancreatitis events, have been quantified meta-analytically, although the causality remains debated [74], and isolated reports of acute pancreatitis temporally associated with tirzepatide have appeared [73]. A history of pancreatitis or symptomatic gallstone disease is a recognized reason to consider switching; however, because the risk appears to be class-related, switching within cannot be a priori assumed to eliminate it [74,75] and current recommendations thus call for complete withdrawal and shift to non-incretin based therapy [76]. Diabetic retinopathy warrants specific attention when switching. An early worsening signal with semaglutide was observed in SUSTAIN-6, confined mainly to patients with pre-existing retinopathy who undergo rapid glycemic improvement [3]; mechanistic data link progression to the magnitude and speed of reduction in HbA1c rather than a direct drug effect [103]. A switch producing abrupt glycemic improvement, for example, from a submaximal GLP-1 RA dose to tirzepatide, could therefore transiently increase risk in susceptible patients with long-standing T2DM, and retinal screening before and after such switches is advisable [3,103]. The dedicated FOCUS trial (clinicaltrials.gov identifier NCT03811561) evaluates the retinal safety of semaglutide prospectively; results are not yet available.

### 8.5. Post-Bariatric Surgery Patients

Weight regain after metabolic and bariatric surgery (MBS) is common and meta-analytic evidence supports the use of GLP-1 RA in this setting, demonstrating significant additional weight loss with an acceptable safety profile [109]. Switching is frequently considered when the initial post-MBS agent produces a plateau or intolerable AEs; altered GI anatomy after procedures such as Roux-en-Y gastric bypass or sleeve gastrectomy may modify tolerability and pharmacokinetics, although dedicated switch trials in post-MBS populations are absent. In the absence of such data, general switching principles, attention to timing, conservative starting doses, and monitoring, should be applied, with increased vigilance for nutritional deficiencies and gastrointestinal symptoms [109].

## 9. Persistence, Discontinuation and Weight Regain

Real-world persistence with incretin-based therapies is substantially lower than in pivotal trials. Routine care analysis consistently shows that many patients with obesity do not receive GLP-1 RAs according to labeled titration schedules, with dose escalation frequently delayed and a large proportion remaining at submaximal doses [16,47]. In T2DM, persistence is similarly suboptimal and delays in reaching maintenance doses are common [48]. Discontinuation is frequent among patients without diabetes, and switching between agents, often a marker of dissatisfaction or inadequate response, precedes discontinuation in a substantial subset [17,18]. Cost and insurance-coverage interruptions are the leading drivers of discontinuation of treatment; survey data indicate that out-of-pocket expense and loss of coverage account for a large share of discontinuations, in addition to GI AEs and supply shortages [79,110]. Delayed escalation itself carries a dual penalty: patients stuck on introductory doses neither achieve the full treatment effect nor, paradoxically, always avoid early AEs that undermine persistence [16,47].

The consequences of stopping therapy are well characterized. In the extension of the STEP 1 trial, participants who withdrawn from 2.4 mg of semaglutide regained approximately two-thirds of their lost weight within one year, and the cardiometabolic benefits retreated to baseline [93]. The SURMOUNT-4 withdrawal design demonstrated that participants who switched from tirzepatide to placebo regained a substantial fraction of lost weight, whereas those who continued tirzepatide maintained and extended their weight loss [95]. STEP 4 similarly showed that continued semaglutide after a 20-week run-in produced further weight loss, while placebo substitution led to regain [94]. Real-world data mirror these experimental findings: discontinuation is followed by progressive weight regain, and the trajectory of regain approximates pretreatment weight over time in a substantial proportion of patients [18,111]. Two complementary 2026 meta-analyzes have quantified this rebound. A systematic review and meta-analysis estimated that after cessation of weight-management medication, weight is regained at an average of approximately 0.4 kg per month, with a projected return to baseline weight within approximately 1.7 years, and that improvements in cardiometabolic markers (HbA1c, fasting glucose, lipids, blood pressure) revert toward pretreatment levels within approximately 1.4 years [112]. A reconstructed aggregate-data Bayesian longitudinal meta-analysis of six semaglutide and tirzepatide withdrawal studies (10 arms, n = 1776) estimated a mean regain of 1.04 kg per month (95% credible interval 0.80–1.29) after cessation of these two agents, with 50% of the initial weight loss regained by 7.5 months and a projected return to baseline by approximately 15 months. After adjustment, regain velocity did not differ between semaglutide and tirzepatide, indicating that the rebound is driven by the magnitude of the initial weight loss rather than by the specific molecule [96]. These figures underscore why switching to a better-tolerated agent, preserving the continuity of incretin-based pharmacotherapy, is preferable to the total abandonment of therapy.

Some patients who discontinue therapy subsequently restart or switch to an alternative agent, suggesting that treatment courses in practice are often episodic rather than continuous [18]; the efficacy implications of such intermittent exposure remain poorly characterized [110,111]. To mitigate weight regain while managing medication costs and long-term exposure, down-titration to the lowest fractional dose that sustains the achieved weight loss (“maintenance microdosing”) is increasingly explored in routine care [101]. Prospective controlled data on such maintenance protocols remain scarce, and the strategy should be regarded as a pragmatic compromise between discontinuation and indefinite maximal dosing rather than an evidence-based standard.

These observations carry a clear clinical message for the changing context. Switching, substituting for another incretin-based agent with continuity of pharmacotherapy, must be conceptually distinguished from discontinuation. Where a switch is motivated by tolerability, cost, or access constraints, preservation of uninterrupted incretin-based treatment is a reasonable therapeutic objective, because even a structured gap in therapy risks initiating the regain trajectory documented in withdrawal studies [93,95,110]. Practical measures include avoiding unnecessary washout intervals between weekly agents with overlapping half-lives, planning switches around supply interruptions in advance, and identifying the most affordable in-class alternative rather than abandoning therapy altogether [79,110]. The frame of obesity as a chronic, relapsing disease requiring long-term treatment, analogous to hypertension or T2DM, helps set patient expectations at the point of switch: the new agent is not a short course, but a continuation of indefinite therapy, and discontinuation should be an actively managed decision rather than a default outcome of side effects or cost [93,110].

## 10. Future Directions

The therapeutic pipeline will reshape the switching landscape within the next several years. Retatrutide, a triple GIP/GLP-1/glucagon receptor agonist, produced weight reductions approaching 24% at 48 weeks in phase 2 testing, numerically greater than the weight losses reported in the pivotal trials of currently approved agents, although this indirect, cross-trial comparison should be interpreted cautiously in the absence of head-to-head data [113]. CagriSema, the fixed combination of the amylin analog cagrilintide with semaglutide, demonstrated weight loss of approximately 23% in REDEFINE-1 and clinically meaningful weight and glycemic benefits in T2DM in REDEFINE-2 [114,115]. Orforglipron, an oral non-peptide small-molecule GLP-1 RA, achieved substantial weight loss in ATTAIN-1 and glycemic efficacy in T2DM in ACHIEVE-1, without the food and water restrictions required for oral peptide semaglutide [45,116]. Amycretin, a unimolecular GLP-1 and amylin receptor agonist, has shown large weight reductions in early-phase trials of both subcutaneous and oral formulations [117,118]. Each new mechanism and formulation will generate new switching questions: transitions from dual to triple agonism, from peptide injectables to oral small molecules, and between agents with overlapping but non-identical receptor pharmacology, for which no pharmacokinetic or clinical conversion evidence yet exists.

Oral small molecules may prove particularly consequential for long-term disease management. The ATTAIN-MAINTAIN trial demonstrated that weight reduction is largely maintained after switching from injectable incretin therapy to oral orforglipron [91]. This injectable-to-oral maintenance paradigm, intensive injectable induction followed by convenient oral continuation, could plausibly reduce discontinuation related to injection burden. Whether it would also reduce cost-driven discontinuation is unknown, as the relative pricing and reimbursement of oral small molecules versus injectables have not yet been established. This sequencing concept should therefore be regarded as a hypothesis requiring prospective evaluation rather than an emerging standard of care [91,116].

Despite this progress, the evidence base for the switch itself remains thin. With the exceptions of SURPASS-SWITCH, which randomized patients on dulaglutide to switch to tirzepatide or dose escalation, and the SWITCH-SEMA program examining transitions to semaglutide, no randomized trial has been designed primarily around a switching strategy, and most practical guidance continues to rest on expert consensus, pharmacometric modeling, and observational data [23,85,89]. Priorities for future research include adequately powered head-to-head switch trials with standardized cross-titration arms; rigorous, trial-derived equivalent-dose evidence to replace extrapolated conversion tables; and validation of precision-medicine predictors, pharmacogenomic markers, responder phenotypes, and early treatment trajectories, that could make switching proactive rather than reactive [55,61,62,119]. Standardized reporting of switcher subgroups within pivotal trials would also materially improve the evidence available to clinicians.

In summary, the coming generation of incretin-based and multi-agonist therapies will multiply both the opportunities and the uncertainties of treatment transitions. The field would be well served by a shift from empiric, consensus-driven switching towards an evidence-based discipline of therapeutic sequencing, in which the choice of the next agent is informed by the patient’s response phenotype, the pharmacological rationale for the transition, and trial-derived guidance on dose conversion and titration. Until such evidence matures, the principles synthesized in this review—continuity of therapy, conservative and individualized titration, and anticipatory management of special clinical situations—offer the most defensible framework for navigating an increasingly crowded and potent pharmacopeia.

## 11. Limitations

Several limitations of this review should be acknowledged. First, as a narrative rather than a systematic review, the literature search and study selection were not governed by a registered protocol, and no formal risk-of-bias or quality assessment of the included studies was performed. Selection bias cannot therefore be excluded. Second, the evidence base is heterogeneous in design, population, indication (T2DM versus obesity) and outcome definition, which precluded quantitative synthesis and complicates cross-study comparisons. Third, dedicated randomized switching trials remain scarce, SURPASS-SWITCH and the flexible-titration pilot study being the principal exceptions, so that several recommendations, particularly for adverse-event-driven switches, shortage-driven substitutions and injectable-to-oral transitions, rest on observational data, pharmacometric modeling and expert consensus and should be regarded as provisional. Fourth, part of the cited evidence is available only as preprints or early reports that have not completed peer review. Finally, the regulatory landscape is evolving rapidly. Jurisdictional indications and dosing statements are accurate as of 1 September 2026 and may change. These limitations qualify the strength of our conclusions, which should be read as a structured interpretation of the best available evidence rather than as definitive guidance.

## 12. Conclusions

Switching between GLP-1 RAs and the dual GIP/GLP-1 receptor agonist tirzepatide has become a routine element of incretin-based pharmacotherapy in T2DM and obesity, occurring in roughly one in four to one in three treated patients, but it remains one of the least standardized acts in the treatment of these conditions. This review aimed to synthesize the pharmacological diversity underlying differential response, evaluate the switching and cross-titration evidence, translate it into a practical algorithm, and delineate evidence gaps. Several conclusions emerge.

First, the substantial molecular heterogeneity of the class in PK/PD, combined with interindividual variability in response, provides a rational basis for switching: failure of one agent neither predicts nor precludes response to another, and real-world and randomized data confirm that switched patients frequently achieve additional glycemic and weight benefit. Second, the evidence, though still limited, has moved beyond the pure opinion of experts. The randomized data now show that switching from dulaglutide to tirzepatide outperforms the escalation of the incumbent agent; GLP-1 RA-experienced, tolerability-proven patients can transition directly to higher-than-initial doses, with preserved tolerability; and flexible, slowed titration substantially reduces GI treatment withdrawal without compromising efficacy. Third, no official dose equivalence between molecules exists, and pharmacometric analyzes demonstrate that milligram doses are not interchangeable, so every transition requires explicit dosing rationale, counseling, and early follow-up rather than reliance on unofficial conversion charts. Fourth, because discontinuation is reliably followed by weight regain, switching should be framed, when clinically appropriate, as a strategy for preserving the continuity of incretin-based therapy when access, cost, or tolerability forces a change of agent.

The most important unmet needs are dedicated, adequately powered switching trials with standardized cross-titration arms, trial-derived dose-equivalence evidence, and validated precision predictors: pharmacogenomic markers, responder phenotypes, and early treatment trajectories that would make switching proactive rather than empirical. Until such evidence matures, the practical message for clinicians is to match the transition strategy to the reason for the switch, respect the pharmacology of both the departing and incoming agent, and plan and follow up with each transition so that a switch does not inadvertently become a discontinuation.

## Figures and Tables

**Table 1:** Pharmacological profiles, maintenance doses, titration schedules and indications by jurisdiction of GLP-1 receptor agonists, the dual GIP/GLP-1 receptor agonist tirzepatide, and the oral small molecule orforglipron.

Molecule	Receptor Targets	Half-Life	Administration (Route/Frequency)	Titration Steps and Maintenance Doses	Approved or Pending Indications by Jurisdiction (T2DM/Obesity/CV Risk)
Exenatide immediate-release	GLP-1R	~2.4 h	s.c., twice daily	5 µg twice daily for 4 weeks → 10 µg twice daily	T2DM
Exenatide extended-release	GLP-1R	steady state ~6–7 weeks (microspheres)	s.c., once weekly	No titration; 2 mg once weekly	T2DM
Lixisenatide	GLP-1R	~3 h	s.c., once daily	10 µg once daily for 14 days → 20 µg once daily	T2DM
Liraglutide	GLP-1R	~13 h	s.c., once daily	T2DM: 0.6 mg for 1 week → 1.2 mg (→1.8 mg if needed). Obesity: 0.6 mg with weekly increments → 3.0 mg	T2DM (with CV risk reduction); obesity at 3.0 mg
Dulaglutide	GLP-1R	~5 days	s.c., once weekly	0.75 mg → 1.5 mg; further escalation to 3.0 mg and 4.5 mg at ≥4-week intervals if needed	T2DM (with CV risk reduction)
Semaglutide s.c.	GLP-1R	~1 week	s.c., once weekly	T2DM: 0.25 mg for 4 weeks → 0.5 mg (→1.0 mg → 2.0 mg if needed). Obesity: 0.25 → 0.5 → 1.0 → 1.7 → 2.4 mg in 4-week steps (optional 7.2 mg weekly for BMI ≥ 30 kg/m^2^)	T2DM (with CV risk reduction); obesity; CV risk reduction in overweight/obesity without diabetes
Semaglutide oral	GLP-1R	~1 week	Oral, once daily (on an empty stomach after ≥8 h of fasting, ≤120 mL water, ≥30 min before intake)	EU formulation: 1.5 mg for 30 days → 4 mg (→9 → 25 or 50 mg if needed)	T2DM (with CV risk reduction)
Semaglutide oral tablets (US)	GLP-1R	~1 week	Oral, once daily (on an empty stomach after ≥8 h of fasting, ≤120 mL water, ≥30 min before intake)	1.5 mg days 1–30 → 4 mg days 31–60 → 9 mg days 61–90 → 25 mg maintenance	Obesity; CV risk reduction in overweight/obesity with established CVD (US)
Tirzepatide	GIPR + GLP-1R	~5 days	s.c., once weekly	2.5 mg for 4 weeks → 5 mg; further 2.5 mg increments at ≥4-week intervals to 10 mg or 15 mg	T2DM (with CV risk reduction); obesity
Orforglipron	GLP-1R	~29–49 h	Oral, once daily (no food or water restrictions)	0.8 mg for ≥30 days; titration steps of 2.5 mg, 5.5 mg, 9.0 mg, 14.5 mg once daily (escalations after at least 30 days); max 17.2 mg once daily	Weight management (US, approved April 2026); weight management and T2DM (UK, authorized August 2026); under EMA evaluation for obesity and T2DM (EU, pending)

Note: Half-lives and pharmacokinetic/pharmacodynamic (PK/PD) characteristics are drawn from class reviews and molecule-specific development reports [33,34,35]; titration and maintenance-dose schedules summarize prescribing information as synthesized in practical guidance and program overviews [20,36]; cardiovascular (CV) indications are based on the respective outcome trials [1,2,3,6]. Approved doses, titration steps and indications are drawn from the current European Medicines Agency (EMA) Summaries of Product Characteristics, the UK Medicines and Healthcare products Regulatory Agency (MHRA) Summary of Product Characteristics, and the US Food and Drug Administration (FDA) prescribing information [28,29,30,31], and vary between jurisdictions. Indications listed as “under evaluation” had not been granted marketing authorization in the respective jurisdiction at the time of writing (September 2026). GLP-1, glucagon-like peptide-1; GLP-1R, GLP-1 receptor; GIP, glucose-dependent insulinotropic polypeptide; GIPR, GIP receptor; MEN 2, multiple endocrine neoplasia syndrome type 2; MTC, medullary thyroid carcinoma; s.c., subcutaneous; T2DM, type 2 diabetes mellitus; BMI, body mass index; CVD, cardiovascular disease; EU, European Union; UK, United Kingdom; US, United States. Regulatory Discrepancy Note: In the United States, most, but not all, agents in this class carry a Food and Drug Administration (FDA) boxed warning regarding the risk of thyroid C-cell tumors, based on rodent carcinogenicity findings, and are contraindicated in patients with a personal or family history of medullary thyroid carcinoma (MTC) or multiple endocrine neoplasia syndrome type 2 (MEN 2). This applies to liraglutide, dulaglutide, semaglutide (subcutaneous and oral), tirzepatide, exenatide extended-release, and orforglipron. The US prescribing information for exenatide immediate-release and lixisenatide does not carry this boxed warning. Notably, the orforglipron label specifies that orforglipron is not pharmacologically active at the rodent GLP-1 receptor and did not produce thyroid tumors in rodents. The warning is retained on a class-wide basis and its human relevance has not been determined [30]. In contrast, the European Medicines Agency (EMA) restricts the absolute contraindication for this class to hypersensitivity to the active substance or excipients, listing thyroid adverse events only under precautions because of the low clinical relevance attributed to the rodent findings in humans.

**Table 2:** Study-level summary of the principal switching, cross-titration, and discontinuation (counterfactual) studies.

Study (Design)	Population	Prior Treatment → Transition Regimen	Comparator	Follow-Up	Principal Outcomes	Key Limitations
Billings 2025, SURPASS-SWITCH (RCT, open-label) + subgroup analysis [23,70]	T2DM, inadequately controlled on dulaglutide	Dulaglutide (lower doses) → tirzepatide, label titration	Dulaglutide escalated to 4.5 mg	40 wk	Greater HbA1c and weight reductions with switching; consistent across subgroups	Single predecessor molecule; open-label; does not inform obesity-dose transitions
Jabbour 2024 (prospective, single-arm) [24]	T2DM on stable GLP-1 RA	Various GLP-1 RAs → tirzepatide 5 mg directly, no re-titration	None	12 wk	HbA1c −0.43%; weight −2.15 kg; GI AEs mostly mild; ~2% AE discontinuation	Uncontrolled; short follow-up; single starting dose
Eldor 2025 (randomized open-label pilot) [25]	Semaglutide-treated patients	16-week flexible titration with intermediate increments	Label 8-week escalation	26 wk	GI-related AEs 2% vs. 19%; equivalent efficacy	Pilot size; open-label; titration, not switch, trial
Tham 2022 (PK/PD simulation) [22]	Virtual T2DM	Semaglutide → dulaglutide 3.0/4.5 mg	Modelled	Simulated	Equal milligram doses not clinically equivalent across molecules	No clinical validation
Overgaard 2019 (exposure–response model) [26]	T2DM (trial-derived)	Various GLP-1 RAs → semaglutide	Modelled	Simulated	Prior GLP-1 RA exposure permits higher starting doses; maintained/improved HbA1c and weight	Model-based; pre-tirzepatide era
Urva 2024 (model simulation) [27]	Virtual T2DM	Semaglutide or dulaglutide → tirzepatide	Modelled	Simulated	Transient PK overlap confirmed; effects follow expected trajectory; supports no-washout timing	Simulation; no clinical outcomes
Jain 2021, REALISE-DM (retrospective RWE) [83]	T2DM	Liraglutide or dulaglutide → semaglutide	None (pre/post)	~6 mo	Improved HbA1c and weight after switch	Retrospective; confounding by indication
Lingvay 2021, EXPERT (retrospective) [84]	GLP-1 RA-experienced T2DM	Various GLP-1 RAs → semaglutide	GLP-1 RA-naïve (descriptive)	~6 mo	Further HbA1c/weight improvements, smaller than in naïve patients	Retrospective; heterogeneous predecessors
Takahashi 2023, SWITCH-SEMA 1 (RCT, open-label) [85]	T2DM (Japan)	Liraglutide or dulaglutide → s.c. semaglutide	Continued prior GLP-1 RA	24 wk	Improved glycemic control and treatment satisfaction	Open-label; single region; older comparators
Iijima 2023 (RCT) [86]	T2DM (Japan)	Liraglutide → semaglutide or dulaglutide	Between-agent	8, 16 and 26 wk	Maintained or improved glycemic efficacy	Small sample; regional
Di Dalmazi 2022 (real-world) [68]	T2DM	Once-weekly GLP-1 RA → another once-weekly GLP-1 RA	Naïve (descriptive)	6–12 mo	Further HbA1c/weight reductions, attenuated vs. naïve	Observational; no standardized regimen
Di Loreto 2022 (retrospective) [69]	T2DM (Umbria)	Various GLP-1 RAs → semaglutide	Naïve initiators	12–18 mo	Switchers improved, less than naïve	Retrospective; single region
Matsushiro 2025 (retrospective) [87]	T2DM (Japan)	Various GLP-1 RAs → tirzepatide at non-initiation doses	None	Short-term	Feasible direct above-initiation-dose switches; preserved tolerability	Retrospective; short follow-up
Barakat 2024 (case series) [88]	T2DM	Various GLP-1 RAs → tirzepatide, pragmatic conversions	None	Variable	Acceptable tolerability and glycemic outcomes	Case series; selection bias
Furusawa 2024, SWITCH-SEMA 2 (RCT, open-label) [89]	T2DM (Japan)	DPP-4 inhibitor → oral semaglutide	Continued DPP-4i	24 wk	Improved glycemic control and weight	Non-incretin predecessor; open-label
Buse 2020, PIONEER 7 extension (open-label extension) [90]	T2DM	Sitagliptin → oral semaglutide	Continued sitagliptin	52 wk total	Additional glycemic and weight improvements	Extension design; non-incretin predecessor
Aronne 2026, ATTAIN-MAINTAIN (RCT, double-blind, phase 3b) [91]	Obesity, after loss on injectables	Injectable semaglutide/tirzepatide → oral orforglipron	Placebo	52 wk	Weight reduction largely maintained after injectable-to-oral switch	Single successor molecule; maintenance setting
Lahey 2025 (retrospective RWE) [92]	T2DM on insulin ± GLP-1 RA	GLP-1 RA → tirzepatide	None	Short-term (weeks)	Rapid, marked insulin dose reduction; hypoglycemia risk without de-escalation	Retrospective; insulin outcomes only
Wilding 2022, STEP 1 ext. (trial withdrawal) [93]	Obesity, no diabetes	Semaglutide 2.4 mg → withdrawal	Off-treatment	52 wk	~Two-thirds of lost weight regained within 1 yr; cardiometabolic benefits reverted	Counterfactual (withdrawal, not switching)
Rubino 2021, STEP 4 (randomized withdrawal) [94]	Obesity, no diabetes	Semaglutide 2.4 mg run-in → placebo	Continued semaglutide	48 wk	Continued therapy: further loss; withdrawal: regain	Counterfactual evidence
Aronne 2024, SURMOUNT-4 (randomized withdrawal) [95]	Obesity, no diabetes	Tirzepatide run-in → placebo	Continued tirzepatide	52 wk	Continued: maintained/extended loss; withdrawal: substantial regain	Counterfactual evidence
Kow 2026 (Bayesian aggregate-data meta-analysis; 6 studies, 10 arms, n = 1776) [96]	Overweight/obesity (one T2DM arm)	Semaglutide or tirzepatide → discontinuation	Pooled off-treatment arms	4–52 wk observed	Regain 1.04 kg/mo (95% CrI 0.80–1.29); 50% of loss regained by 7.5 mo; baseline ~15 mo; no drug-specific rebound	Complete overlap with source review; extrapolation beyond 52 wk

AE, adverse event; CrI, credible interval; DPP-4i, dipeptidyl peptidase-4 inhibitor; GI, gastrointestinal; GLP-1 RA, glucagon-like peptide-1 receptor agonist; HbA1c, glycated hemoglobin; mo, months; PK/PD, pharmacokinetic/pharmacodynamic; RCT, randomized controlled trial; RWE, real-world evidence; T2DM, type 2 diabetes mellitus; vs, versus; wk, week.

**Table 3:** Practical considerations when switching between GLP-1 RAs and tirzepatide.

Clinical Scenario	Suggested Approach	Evidence Level/Source
Efficacy-driven switch from a GLP-1 RA to tirzepatide	Administer tirzepatide when the next dose would be due; direct start at 5 mg is reasonable in stable, tolerability-proven patients	RCT (SURPASS-SWITCH) [23,70]; prospective switch study [24]
AE-driven switch	Re-titrate from the lowest dose of the new agent; slow or flexible escalation	Expert consensus [19,21]; RCT (flexible titration) [25]; pooled AE analysis [99]
Switch due to shortage or loss of access	Document prior dose and treatment gap; re-initiate approved product with dose re-titration if the gap is prolonged; avoid compounded products	Practical strategies and systematic review [77,78]; ADA statement [80]
Injectable to oral switch	Respect SNAC fasting conditions for oral semaglutide; consider maintained efficacy data when switching from injectables	Trials [89,90,91]; PK studies [42,44]
Switch in insulin- or sulfonylurea-treated patients	Proactively de-escalate insulin/sulfonylurea; intensify glucose monitoring during the first weeks	Real-world study [92]; consensus guidance [20]
GLP-1 RA-experienced versus GLP-1 RA-naïve	Experienced, tolerability-proven patients may start above the nominal initiation dose; naïve patients should follow standard label titration	Real-world and model-based evidence [24,26,68,87]

GLP-1 RA, glucagon-like peptide-1 receptor agonist; RCT, randomized controlled trial; AE, adverse event; GI, gastrointestinal; ADA, American Diabetes Association; PK, pharmacokinetics.

**Table 4:** Provenance of each algorithmic recommendation by strength and type of supporting evidence.

Algorithm Element	Recommendation	Evidence Type (Sources)
Step 1—Classify the switching trigger	Trigger determines successor agent, starting dose, and titration speed	Expert consensus [19,20,21]; author interpretation
Step 2—Safety prerequisites	Contraindication and AE-history review; retinopathy caution with rapid glycemic improvement	Expert consensus [19,20,21]; indirect trial evidence [3,103]
Step 2—Background therapy	Anticipatory de-escalation of insulin/sulfonylureas with intensified monitoring	Observational real-world data [92]; expert consensus [20,21,71]
Step 3—Efficacy-driven switch, tolerability-proven patient	Direct switch at a higher-than-initial dose (tirzepatide 5 mg)	Direct switching evidence: RCT [23,70]; prospective study [24]; real-world series [87,88]
Step 3—AE-driven switch	Restart at the lowest dose; slow or flexible escalation	Expert consensus [19,20,21]; randomized pilot [25]; pooled AE analysis [99]
Step 3—Switch after a treatment gap	After a gap exceeding ~2–3 half-lives, treat as treatment-naïve and re-titrate	Expert consensus [20,21]; PK rationale [27]; author extrapolation
Step 4—Timing	Administer the new agent when the next dose of the previous agent is due; no washout	Expert consensus [19,20,21]; pharmacometric simulation [26,27]
Step 4—Dose conversion	No milligram equivalence; avoid unofficial conversion charts	Model-based analyzes [22,98]; proposed equivalent-dose table [20]; author interpretation
Step 5—Staged monitoring schedule	Weeks 4 and 8: assess safety, GI tolerability, glucose, and adjust concomitant medication; week 12 (≈ month 3): initial efficacy assessment and primary decision node (HbA1c, weight trajectory, adherence, dose exposure, tolerability); week 16: conditional assessment if dose titration ongoing or month-3 response equivocal; month 6: reassess only provisional delayed responders (limited early weight loss despite meaningful glycemic improvement)	Indirect evidence: trajectory analyzes [54,59]; responder syntheses [55,58]; author synthesis
Step 6—Titration strategy	Flexible/slowed titration when efficacy plateaus but AEs limit escalation	Randomized pilot [25]; pooled analysis [99]; flexible-titration RCT [100]
Injectable-to-oral maintenance transition	Switch to oral agent feasible in pretreated patients	Direct: randomized trial [91]; label conversion tables [28,29]

AE, adverse event; GI, gastrointestinal; HbA1c, glycated hemoglobin; PK, pharmacokinetic; RCT, randomized controlled trial.

## Data Availability

No new data were created or analyzed in this study. Data sharing is not applicable to this article.

## Abbreviations

The following abbreviations are used in this manuscript:

ADA	American Diabetes Association
AE	Adverse event
BMI	Body mass index
BW	Body weight
CrI	Credible interval
CV	Cardiovascular
CVD	Cardiovascular disease
DPP-4	Dipeptidyl peptidase-4
DPV	Diabetes-Patienten-Verlaufsdokumentation
EMA	European Medicines Agency
EU	European Union
FDA	Food and Drug Administration
GI	Gastrointestinal
GIP	Glucose-dependent insulinotropic polypeptide
GIPR	Glucose-dependent insulinotropic polypeptide receptor
GLP-1	Glucagon-like peptide-1
GLP-1 RA	Glucagon-like peptide-1 receptor agonist
GLP-1R	GLP-1 receptor
HbA1c	Glycated hemoglobin
MACE	Major adverse cardiovascular events
MBS	Metabolic and bariatric surgery
MHRA	Medicines and Healthcare products Regulatory Agency
MEN 2	Multiple endocrine neoplasia syndrome type 2
MTC	Medullary thyroid carcinoma
PAM	Peptidyl-glycine alpha-amidating monooxygenase
PD	Pharmacodynamics
PK	Pharmacokinetics
RCT	Randomized controlled trial
RWE	Real-world evidence
s.c.	Subcutaneous
SNAC	Sodium N-(8-[2-hydroxybenzoyl]amino) caprylate
SPAQI	Society for Perioperative Assessment and Quality Improvement
T2DM	Type 2 diabetes mellitus
UK	United Kingdom
US	United States
